# Fitness consequences of chronic exposure to different light pollution wavelengths in nocturnal and diurnal rodents

**DOI:** 10.1038/s41598-022-19805-1

**Published:** 2022-10-01

**Authors:** Hagar Vardi-Naim, Ava Benjamin, Tali Sagiv, Noga Kronfeld-Schor

**Affiliations:** School of Zoology, Tel Aviv University, Tel Aviv, Israel

**Keywords:** Conservation biology, Ecophysiology

## Abstract

Use of artificial at night (ALAN) exposes the world to continuously increasing levels and distribution of light pollution. Our understanding of the adverse effects of ALAN is based mostly on observational or laboratory studies, and its effects are probably underestimated. Demonstration of direct experimental fitness consequences of ALAN on mammals is missing. We studied the effects of chronic light pollution at different wavelengths on fitness and glucocorticoid hormone levels under semi-natural conditions in two closely related species: the nocturnal common spiny mouse (*Acomys cahirinus*) and the diurnal golden spiny mouse (*Acomys russatus*). Our results clearly demonstrate the adverse effects of ALAN exposure on the fitness of both nocturnal and diurnal species, manifested by changes in cortisol levels and reproductive timing, reduced reproductive output and reduced survival, which differed between species and wavelengths. In *A. russatus* exposure to blue ALAN had the strongest effect on fitness, followed by white and yellow ALAN exposure. In *A. cahirinus* the results are more complex and suggest it suffered from the combined effects of ALAN and competition. Our research shows that light pollution presents a real threat to both nocturnal and diurnal species, affecting the species fitness directly and through interspecific interactions. Worryingly, these effects are probably not limited to spiny mice. The clear adverse effects we documented, as well as the differences between wave lengths, contribute to our ability to present science-based recommendations to decision makers regarding the use of artificial light at night. Such information and guidelines are highly important nowadays when lighting systems are being replaced to promote energy efficiency.

## Introduction

The extensive and increasing use of artificial at night (ALAN) exposes the world to continuously increasing levels and distribution of light pollution. The new world atlas of light pollution^1^ portrays an unsettling reality, in which 99% of the US population and 80% of the world’s population live under light-polluted skies, and it is estimated that in areas where light pollution has not reached saturation, the increase in ALAN radiance and extent is 2–6% per year^2–4^.

Light pollution has attracted increasing scientific attention over the last two decades. Though it was considered to have a limited spatial extent compared to more familiar anthropogenic interferences, such as climate change and plastic pollution, it is now accepted that ALAN effects are much more pervasive than previously considered, and that it is becoming a major threat to biodiversity^2,5–8^. ALAN affects all living organisms at the molecular, physiological, behavioral, and ecological level. For example, it was shown to disrupt foraging and feeding behavior, alter biological rhythms, cause mistimed reproductive behavior, and increase stress hormone levels (e.g.^9–20^), and it is clear that actions must be taken to mitigate its effects. However, to design effective mitigation measures we must better understand the adverse effects of light pollution, which are currently based mostly on observational field or laboratory studies, mainly focusing on changes in species behavior and physiology^5,8,20,21^.

Length of photoperiod (day length) is a reliable indicator of time of year and serves as a cue for seasonal acclimation^18,22^. However, the extensive use of artificial light disrupts the clear delineation between day and night, and the reliability of photoperiod is compromised. As a result, seasonal acclimation and seasonal reproduction may be compromised, reducing both survival and reproductive success. Disruption of the natural light–dark cycle by ALAN may cause physiological and behavioral changes (reviewed by Bumgarner and Nelson^23^). ALAN exposure changed the endocrine system, and glucocorticoid secretion in particular, in bird^9,24,25^ and rodent species^26^, affected sexual behavior and fertilization success in common toads, *Bufo bufo*^27^, and advanced avian reproductive physiology^11^, and there is even an experimental field study on the effects of ALAN at different wavelength on physiology, behavior and ecology of species^9,28,29^. Yet, to the best of our knowledge, no study tested the effects of ALAN on survival and offspring number, which are the direct measures of fitness, and experimental fitness consequences of ALAN exposure on rodents have yet to be examined. Moreover, to promote science-based policies regarding the outdoor use of light at night, the effects of different wavelengths of ALAN at relevant intensities should be compared. To close this knowledge gap, we chose to study the effects of chronic light pollution on fitness (survival and reproduction) under semi-natural conditions in two closely-related, coexisting rodent species with opposite activity patterns: the nocturnal common spiny mouse (*Acomys cahirinus*) and the diurnal golden spiny mouse (*Acomys russatus*), which were chronically exposed to ALAN at different wavelengths with relevant intensity.

## Materials and methods

### Experimental design

Experiments took place at the Zoological Research Garden at Tel Aviv University, were we used outdoor enclosures to test the long-term effects of ALAN under semi-natural conditions on two species of spiny mice; the diurnal *Acomys russatus* and the nocturnal *A. cahirinus*. Experiments were conducted for 6–8 months in two consecutive years: the first repetition started in November 2018 and ended in July 2019. The second repetition started on November 2019 and ended in June 2020.

All experiments were carried out in accordance with the Israeli Ministry of Health guide for the care and use of laboratory animals and all experimental protocols were approved by the Tel-Aviv University Institutional Animal Care and Use Committee (IACUC protocol approval number 04-18-056, 04-19-031).

### Enclosures

**Figure 1:**
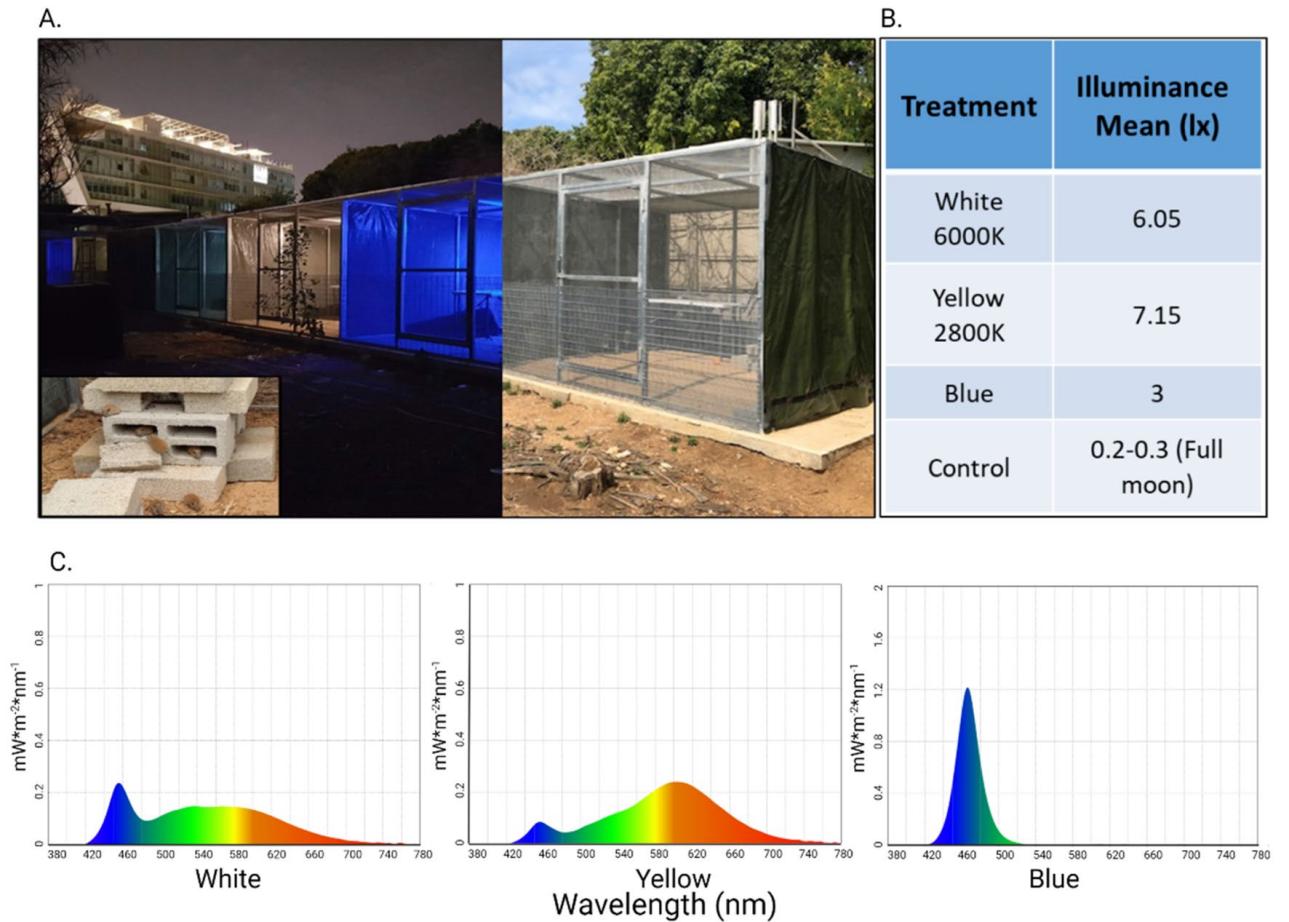
(**A**) Enclosure set-up, in daylight and at nighttime. Eight enclosures arranged side by side in a row were built to house spiny mice under semi-natural conditions. Hollow cement blocks were used as shelters for both species. From right to left: enclosures in day light, enclosures at night with blue, yellow and white light. Control enclosures remained dark. (**B**) Light treatment illuminance and (**C**) spectral distribution.

An enclosure complex of eight 3X3X3-meter metal mesh cages was built to prevent mice from escaping and protect them from predation, while allowing exposure of natural sunlight, moonlight and weather conditions. The rear part of the enclosures was covered with a tin roof to protect the mice from overheating and flooding. Enclosures were separated using white tin sheets to prevent light penetration and interactions between mice from neighboring enclosures. Hollow cement blocks were stacked to form housing structures for the mice (Fig. 1). We divided individuals of both species into four treatments, two experimental enclosures each. Six enclosures were exposed to ALAN in different wavelengths (two enclosures/wavelength) translating to different colors: white light (420–740 nm) which is most commonly used and contains a peak in the blue range, yellow (warm white) light (430–740 nm) which is recommended for use outdoors by the nature conservation bodies, and blue light (420–520 nm), known to affect the biological clock^30^, which is the main difference between the other two treatments. Light measurements were carried out using C-700 Spectrometer by Sekonic (see Fig. 1 for spectrum and intensity measures). The other two enclosures were not exposed to ALAN and served as controls (half-moon light level ca 0.1 lx). Each enclosure contained 12 individuals (6/species) with equal numbers of males and females from each species. To follow the natural photoperiod, lights were automatically turned on 30 min before sunset and turned off 30 min after sunrise using an electric timer adjusted to natural photoperiod every two weeks. The LED lamps (Product Nos. G334BL, G334Y, and G334WW, Eurolux, South Africa) were hung from the center of the ceiling in each enclosure, 3 m above the ground. Animals were supplied with nesting material, rodent pellets (Product No. 2018SC, Teklad Global, USA) and water ad libitum.

### Animals

A total of 247 animals took part in the experiment (n_*A. russatus*_ = 117; n_*A. cahirinus*_ = 130). All mice were taken from the spiny mouse colony of the Zoological Research Garden at Tel Aviv University and were between six months and one year old at the beginning of the experiment. The animals were tagged with RFID chips for individual recognition (Product No. 040999, MS Schippers, The Netherlands) and were free to roam their enclosures and engage in social interactions. For physiological measurements and population evaluation the mice were captured once every other week using Sherman traps. Sample size varied between tests, as detailed below.

### Population size and reproductive output

During the experiments we had unexpected mortality. Mortality date was determined by the date the corpse was found or estimated by failure to trap the individual over time. In the latter cases, date of death was determined as the date of first trapping failure. Newborns were trapped and documented and in most cases were removed from the enclosure. In case of mortality in the enclosure, pups born in the same enclosure were tagged with RFID and left in the enclosure in order to maintain the population size and structure as in the beginning of the experiment, with 6 mice from each species, equal number of males and females.

### Cortisol measurements

To examine whether light pollution affects cortisol levels, traps were set 6–8 h before the beginning of activity time of each species in three sessions: March and June 2019 and December 2019 (only during the second year of experiment, detailed in the Supplementary Material Table S1). Feces were collected from the trap up to 8 h from setup to get the baseline cortisol concentration. Fecal cortisol follows a daily rhythm, and elevation of cortisol level as a response to stress (such as entering a trap) can be detected 12 h after the encounterencounter^31^. Feces were kept in – 20 °C until further analysis could be conducted. ELISA kits (ARBOR ASSAYS catalog number K003-H1W/H5W) were used for the analysis. Sample preparation and analysis was conducted according to the steroid Solid Extraction Protocol of the manufacturer, using the given commercial cortisol sample for standard curve. Since most fecal sample weight was low (> 0.2 g), we normalized the data using the following equation *[Assay Concentration (i.e., pg/mL) ÷ (Evaporation Vol (mL) ÷ 0.5)] / [Feces weigh (g) /Evaporated ethanol (mL)* = *Analyte unit (i.e., pg/gm) fecal solid.* Intra assays CV was 15% while inter assay CV was less than 10%. We have not determined extraction efficiency and recovery, but used a kit that is widely used on other mammalian species including rodents^32,33^. Extraction efficiency and recovery in this method using another kit was conducted previously^31^.

### Statistical analysis

Statistical analysis was done using R software (version 4.0.2) and Excel software (Office 2016), as detailed bellow. Differences between groups were considered significant if *p* < 0.05.

#### Survival probability

**Figure 2:**
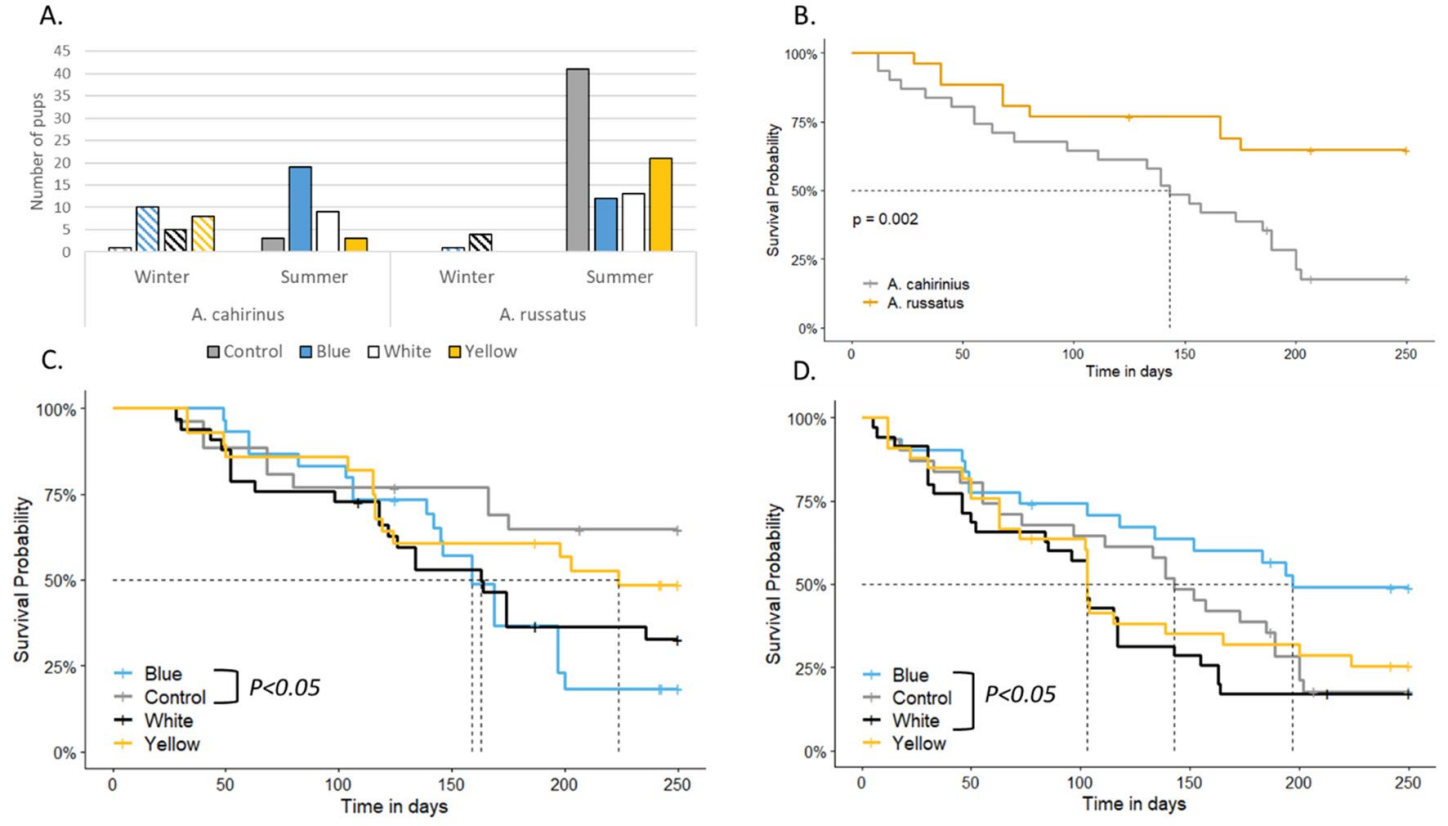
Population data: (**A**) newborns across all treatments for 2 years, divided by season. For more information regarding number of females in each treatment and season, see Supplementary Material Table S2; (**B**) survival probability of the two species from the control group; (**C**) survival probability of *A. russatus* across treatments; (**D**) survival probability of *A. cahirinus* across treatments. Dashed lines show the median survival probability of each group.

We tested the effect of light pollution on the survival probability using Kaplan–Meier analysis^34^. A survival curve was calculated for each treatment with time of exposure in days, applying only to the adults of the enclosure, or pups that were born in the enclosure before light treatment had begun and left at the enclosure to keep sample size. Comparison of survival curves was done by a Post-Hoc Tukey test, using the packages *survival* and *survminer* in Rstudio. Survival rates differed between species (p < 0.05, Fig. 2B); therefore, analysis was done for each species separately.

#### Newborns

Probability that ALAN affects the reproductive success of each species throughout the year, was assessed using Chi Square goodness of fit, under the assumption that reproductive success should be even across enclosures. Grouping the data by seasons was made to emphasize the mistiming of reproductive activity, and not for statistical analysis due to small sample size. Winter includes all pups born from November to February; summer- includes all pups born from March to July.

#### Cortisol

Cortisol concentration in the feces can be affected by species, sex, and season, among many other factors. Feces collected in summer (March and June) were combined to increase sample size, after making sure that cortisol concentration is not significantly different between these months (Wilcoxon rank sum test). Cortisol concentration data were highly skewed and did not distribute normally; therefore, analysis was done using *lme4* package in R, with a Generalized Linear Model (GLM) using gamma distribution. The June data set was partially composed of samples collected from animals that appear in March data set. To overcome this pseudo-replication while keeping the sample size, we used a nested model, where the animal ID and the month of collection are random effects, and light treatment is fixed. Treating the number of enclosures as random effect had no impact on the model results and therefore was not included in the model. The study is reported in accordance with ARRIVE guidelines.

## Results

### Survival probability

In general, *A. cahirinus* survival probability was lower compared to *A. russatus* even when not exposed to ALAN (Fig. 2B, *P* = 0.002). Survival of the diurnal *A. russatus* was significantly lower when exposed to blue light compared to controls (Fig. 2C, *P* = 0.029). *A. cahirinus* exposed to blue ALAN had a significantly higher survival probability compared to *A. cahirinus* exposed to white ALAN (Fig. 2D, *P* = 0.049). Two mortality incidents worth noting occurred under white light treatment in two different enclosures, the first occurred in January 2020 and the second in March 2020. The gap in physical location and time, eliminates the possibility that sudden death is related to the enclosure itself or that it was caused by the same reason. These incidents influenced overall mortality rates.

Despite mortality incidences, mortality rates did not differ significantly between years in both species and therefore years were combined (P_cahirnius_ = 0.27, P_russatus_ = 0.17).

### Reproduction

Reproductive behavior was significantly influenced by exposure to ALAN in both species. Equal distribution of reproductive success (*f*_*e*_ = 0.25) was refuted for both species (*df* = 3, *P* < 0.001, Supplementary Table S2). Total number of pups over the experiments was highest in *A. russatus* controls, and in *A. cahirinus* exposed to blue light. Controls of both species were reproductively active during the summer except for one *A. cahirinus* that was born in the winter (Fig. 2A). When exposed to ALAN, *A. cahirinus* were reproductively active all year including winter months. Reproduction during winter was also evident in *A. russatus* exposed to white and blue ALAN, although to a lower extent.

### Cortisol

**Figure 3:**
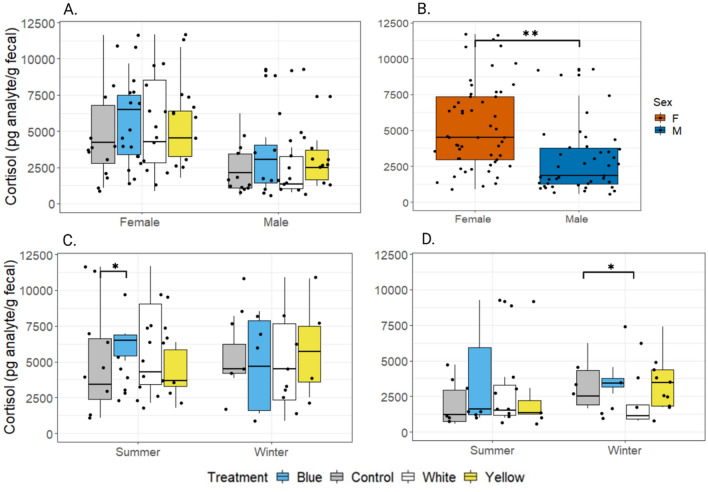
Fecal cortisol concentration of *A. russatus* exposed to different treatments. (**A**) Cortisol concentration in females and males in winter and summer; (**B**) cortisol differences between sexes in all *A. russatus* combined (**P < 0.001); (**C**) cortisol concentration in females in the summer and winter (*P < 0.05); (**D**) cortisol concentration in males in the summer and winter.

Fecal cortisol concentration in female *A. russatus* were significantly higher than males (Fig. 3, *P* < 0.001). Therefore, results were analyzed separately for each sex. Males exposed to blue light at night had a marginally significantly higher level of cortisol compared to controls (Fig. 3, *P* = 0.057). In summer, *A. russatus* females exposed to blue light had significantly higher levels of cortisol compared to controls, while cortisol levels of females exposed to white light were almost significantly different from control (Fig. 3, *P*_blue_ = 0.02, *P*_white_ = 0.055). In winter, cortisol levels of *A. russatus* males exposed to white light were significantly lower compared to controls (Fig. 3, *P* = 0.02).

In *A. cahirinus* there were no differences in fecal cortisol level between groups (Supplementary Materials Fig. S1).

## Discussion

Our experiments resulted in a clear demonstration of the adverse effects of ALAN exposure on the fitness of both nocturnal and diurnal species, either directly or by affecting interspecific interaction. These effects were manifested by reduced survival rates and reproductive success. Mortality rates of diurnal *A. russatus* were significantly higher when exposed to blue ALAN, compared to control groups (Fig. 2C). While the controls did not reach the median of survival until the end of the experiment, *A. russatus* exposed to white and blue ALAN reached the median at similar times (after 160 days of exposure), and yellow-light exposed *A. russatus* reached the median 2 months later (Fig. 2C). These results suggest that the blue, short wavelength light (420–520 nm), which is also included in the white light but almost absent in the yellow light, had an adverse effect on the mice. Blue light is known to have the largest effect on the eye's intrinsically photosensitive, melanopsin containing retinal ganglion cells (ipRGCs) which results in the direct suppression of melatonin and influences biological rhythms^30^. Light intensities as low as 0.028 lx (monochromatic blue light) and 0.3 lx (white light) are capable of suppressing melatonin in rodents and birds, respectively^21^. Therefore, we suggest that our results indicate an involvement of biological rhythms in the effect.

The results of nocturnal *A. cahirinus* are more complex. Survival rates of *A. cahirinus* were generally low, but significantly higher under blue compared to white ALAN exposure. We suggest that it suffered from competition in addition to the ALAN effect; mortality rates in the control groups were significantly higher in *A. cahirinus* compared to *A. russatus* (Fig. 2B, *P* = 0.002), and we suggest that when *A. russatus* survival rates decreased significantly (under blue ALAN exposure), competition pressure reduced as a result survival rates of *A. cahirinus* were significantly higher compared to white ALAN exposure, where competition was presumably more intense. We further suggest that the combined effects of competition and ALAN exposure impacted all the other results we obtained for *A. cahirinus*, including reproductive success and fecal cortisol metabolites levels (see below).

This effect of competition may seem surprising at first, as it seems to contradict the consistent observation in the wild indicating that the nocturnal *A. cahirinus* (which is smaller) competitively excludes *A. russatus* from nocturnal to diurnal activity^35–43^. However, the mechanism of exclusion is resource (food) mediated^43^, and we have previously shown that in direct confrontations *A. russatus* is more aggressive than *A. cahirinus*^44^. Under the current experimental conditions, where food was available ad libitum, we assume there was no competition for food, and suggest that the more aggressive *A. russatus* outcompeted, increased the stress levels, and reduced survival rates and reproductive success of *A. cahirinus.* To test this hypothesis, we are now repeating this experiment while housing the two species in separate enclosures.

The effect of ALAN on reproductive success was striking (Fig. 2, Supplementary Table S2). In their natural habitat, both species breed during summer: young individuals of *A. cahirinus* are observed from February until September, and young *A. russatus* are observed from April to July^45^. While the reproductive activity in both species was affected by ALAN, the nature of their responses was different. *A. russatus* reproductive success was strongly affected by ALAN: the total number of pups of *A. russatus* exposed to all tested wavelengths significantly decreased by at least half compared to the control group (Supplementary Table S2). Yet, in all treatments, reproduction appeared almost only during summer, which is the reproductive season of both species^45^.

In *A. cahirinus* we again see a combined effect of ALAN exposure and interspecific competition, and a different effect of ALAN: the total number of pups of *A. cahirinus*, which was lower than that of *A. russatus* seems to be affected mostly by competition. Reproductive output of *A. cahirinus* is normally higher than that of *A. russatus*^46,47^. Yet, it had a low number of pups in the control group where *A. russatus* thrived most and a high number of pups in the enclosures where *A. russatus* had low survival rates and fewer pups (blue treatment). Moreover, ALAN resulted in loss of seasonality of reproduction in *A. cahirinus*, which was reproductively active year-round under all ALAN exposed enclosures as opposed to the control group, whose reproductive seasonality corresponded to their natural reproductive timing. In long day breeders like *Acomys*, seasonal reproduction is timed by day length, so we conclude that for *A. cahirinus* and somewhat for *A. russatus*, exposure to any light pollution at all tested wavelengths led to a false perception of summer day length and resulted in the loss of seasonality and continuous reproduction*.* Yet, overall reproduction (total number of offspring) was negatively affected by exposure to ALAN.

An effect of light pollution on reproductive timing was previously described^48–50^, but these studies mostly focused on physiological reproductive state and did not measure reproductive output. Our experimental enclosures allowed us to continuously measure reproductive output and demonstrate the negative effect of ALAN on reproduction. However, our result may underestimate the actual effect under natural conditions; unlike natural conditions, the mice in our enclosures had ad libitum access to food and water. It is possible that in the wild, pups born during the winter, when temperatures are low and arthropod (*Acomys* preferred food) availability is lowest^43,51^, and when spiny mice use torpor to reduce energy expenditure^38,52–54^, would have low survival rates, further decreasing fitness.

The effect of ALAN on all measured parameters may be mediated by two hormones—melatonin and glucocorticoids (GC). Daily and seasonal timing cues rely on melatonin secretion, which plays a key role in the biological regulation of daily and seasonal rhythms. Melatonin secretion is confined to the night by the circadian clock and is inhibited by light in both diurnal and nocturnal mammals^55,56^. Therefore, light at night can reduce or stop melatonin secretion, and hence disrupt the natural cycle of all downstream biochemical and physiological processes influenced by melatonin. In the current study we did not measure melatonin secretion, and its role in the observed effects of ALAN in this setup remain to be studied.

We did measure the effect of ALAN on *Acomys* main glucocorticoid—cortisol^31^.We found that exposure to blue and white light at night increased the baseline GC levels of diurnal *A. russatus* (Fig. 3C,D). GC are involved in various processes, acting as neurotransmitters and neuromodulators, activating and regulating numerous processes related to stress response and homeostasis as well as synchronization of peripheral clocks^57,58^. Consistent with our data (Fig. 3B), cortisol normally exhibits a dimorphic difference with higher concentration of GCs among females^59^. Cortisol follows a daily rhythm with tendency to reach high concentration in the blood at or just before awakening time, and decreases during active hours^60^. *Acomys* cortisol daily pattern in fecal material is delayed by12 hours compared to blood concentration, with high concentration of fecal cortisol at 21:00 for *A. russatus* and 16:00 for *A. cahirinus*^31^. Setting the traps and feces collection was done in the beginning of the activity time of each species, representing 12 h earlier, meaning the trough of the daily pattern. Therefore, elevated cortisol levels may indicate chronic stress of the individual or a shift in the circadian pattern of GC.

Cortisol also displays a seasonal pattern in some mammals, which is associated with reproductive activity^61,62^ and metabolic adjustment to changes in energy demand and thermoregulation^63,64^. To distinguish between seasonality in cortisol and the reaction to light pollution, we compared the cortisol levels of each group (species and sex) in each season. We found that in the summer, cortisol levels of *A. russatus* females exposed to blue ALAN were significantly higher (*P* < 0.05) and marginally significant in females exposed to white light (*P* = 0.055) compared to controls (Fig. 3C). Male *A. russatus* exposed to white light in winter had significantly lower levels of cortisol compared to controls (Fig. 3D). Under free-living and semi-natural conditions, spiny mice of both species have lunar cycles in fecal cortisol metabolite levels, with increased levels during moonlit nights^31^ as well as in response to artificially increasing light levels at night to moon levels^65^. Full moon light levels (natural or artificial) also resulted in reduction of activity levels, foraging and food consumption in both species^31,65,66^, and increased inter-specific aggressive interactions in *A. cahirinus*^65^. However, these high cortisol levels were temporary and showed a lunar cycle, while in the current experiment the exposure to ALAN and its effect on cortisol levels are chronic. High or low levels of cortisol may indicate that exposure to ALAN leads to an unbalanced activation of HPA axis with regards to season. Chronic dysregulation of the HPA axis is known to have many adverse physiological consequences and may results in a weakened immunity response^67^ which leaves the individual more susceptible to infectious diseases and parasites^68^, and may explain the higher mortality rates in the groups exposed to blue and white ALAN.

In *A. cahirinus* we found no significant changes in fecal cortisol levels between males and females, treatments, or seasons (Supplementary Fig. S1). We suggest that cortisol levels of all *A. cahirinus* were consistently high in this experiment as a result of competition with *A. russatus*, which masked the effects of ALAN.

We hypothesize that in *A. russatus*, blue and white light increased basal level of cortisol, activated the HPA axis and resulted in the inhibition of reproductive activity. The response to yellow ALAN was weaker in all parameters. The results for *A. cahirinus* are again more complex. Reproductive output of *A. cahirinus* is normally higher than that of *A. russatus*^46,47^. Yet, in our experiment, the total number of *A. russatus* pups was almost double that of *A. cahirinus*. This result, together with the higher mortality rate of *A. cahirinus* supports our hypothesis that *A. cahirinus* suffered from a combined effect of competition and ALAN exposure. This hypothesis also suggests that cortisol levels were chronically high in all *A. cahirinus* groups, and therefore we did not find any effect of sex, season, or treatment as we did in *A. russatus.*

Exposure to blue and white, and to a lesser extent yellow ALAN, changed the interspecific interactions between the species and resulted in population decline and reduced fitness in both species. Such conditions could promote the introduction or spread of alien invasive species that are, in many cases, less sensitive to illumination^65,69–72^.

## Conclusions

Our research clearly shows that light pollution presents a real threat to both nocturnal and diurnal species, affecting species' fitness directly as well as through interspecific interactions. Exposure to ALAN, which masks cyclic environmental light cues has severe consequences, influencing physiological and behavioral processes and ecological interactions that are crucial for the survival and fitness of the species. As a result, ALAN may as a threat to the stability of natural environments, as it alters theehaveor and survival of species in an unequal manner which can influence community structure. Such effects on species fitness can compromise the health of the ecological system, eventually resulting in its collapse. Worryingly, there is no reason to assume the effects are specific to spiny mice.

We also found that the effects differ between wavelengths; blue light had the worst effect, followed by white, and yellow light had the weakest effect, which was still significant. Such findings are highly important for planning mitigation actions. It supports our hypothesis that in mammals, blue and white light have the worst effect on fitness, and yellow light is preferable when illumination is necessary.

## Supplementary Information

Supplementary Information.

## Data Availability

The datasets used and/or analyzed during the current study are available from the corresponding author upon reasonable request.

## References

[1] Falchi F (2016). The new world atlas of artificial night sky brightness. Sci. Adv..

[2] Holker F, Wolter C, Perkin EK, Tockner K (2010). Light pollution as a biodiversity threat. Trends Ecol. Evol..

[3] Kyba C, Mohar A, Posch T (2017). How bright is moonlight?. Astron. Geophys..

[4] Hölker F (2010). The dark side of light: A transdisciplinary research agenda for light pollution policy. Ecol. Soc..

[5] Sanders D, Frago E, Kehoe R, Patterson C, Gaston KJ (2021). A meta-analysis of biological impacts of artificial light at night. Nat. Ecol. Evol..

[6] Gaston KJ, Bennie J, Davies TW, Hopkins J (2013). The ecological impacts of nighttime light pollution: A mechanistic appraisal. Biol. Rev..

[7] Gaston KJ, Bennie J (2014). Demographic effects of artificial nighttime lighting on animal populations. Environ. Rev..

[8] Gaston KJ, Visser ME, Hoelker F (2015). The biological impacts of artificial light at night: The research challenge. R. Soc. Philos. Trans. Biol. Sci..

[9] Ouyang JQ (2016). Stressful colours: Corticosterone concentrations in a free-living songbird vary with the spectral composition of experimental illumination. Biol. Lett..

[10] Ouyang JQ, Davies S, Dominoni D (2018). Hormonally mediated effects of artificial light at night on behavior and fitness: Linking endocrine mechanisms with function. J. Exp. Biol..

[11] Dominoni D, Quetting M, Partecke J (2012). Artificial light at night advances avian reproductive physiology. Proc. Biol. Sci..

[12] Ayalon I (2021). Coral gametogenesis collapse under artificial light pollution. Curr. Biol..

[13] Ayalon I, de Barros Marangoni LF, Benichou JI, Avisar D, Levy O (2019). Red Sea corals under Artificial Light Pollution at Night (ALAN) undergo oxidative stress and photosynthetic impairment. Glob. Change Biol..

[14] Amichai E, Kronfeld-Schor N (2019). Artificial light at night promotes activity throughout the night in nesting common swifts (Apus apus). Sci. Rep..

[15] Kronfeld-Schor N (2021). Drivers of infectious disease seasonality: Potential implications for COVID-19. J. Biol. Rhythms.

[16] Kronfeld-Schor N, Visser ME, Salis L, van Gils JA (2017). Chronobiology of interspecific interactions in a changing world. Philos. Trans. R. Soc. Lond. B.

[17] Kronfeld-Schor N (2013). Chronobiology by moonlight. Proc. R. Soc. B.

[18] Stevenson TJ (2015). Disrupted seasonal biology impacts health, food security and ecosystems. Proc. R. Soc. Lond. B..

[19] Kaniewska P, Alon S, Karako-Lampert S, Hoegh-Guldberg O, Levy O (2015). Signaling cascades and the importance of moonlight in coral broadcast mass spawning. eLife.

[20] Liu JA, Meléndez-Fernández OH, Bumgarner JR, Nelson RJ (2022). Effects of light pollution on photoperiod-driven seasonality. Horm. Behav..

[21] Grubisic M (2019). Light pollution, circadian photoreception, and melatonin in vertebrates. Sustainability.

[22] Stevenson TJ, Prendergast BJ (2015). Photoperiodic time measurement and seasonal immunological plasticity. Front. Neuroendocrinol..

[23] Bumgarner JR, Nelson RJ (2021). Light at night and disrupted circadian rhythms alter physiology and behavior. Integr. Comp. Biol..

[24] Mishra I (2019). Light at night disrupts diel patterns of cytokine gene expression and endocrine profiles in zebra finch (Taeniopygia guttata). Sci. Rep..

[25] Grunst ML (2020). Early-life exposure to artificial light at night elevates physiological stress in free-living songbirds. Environ. Pollut..

[26] Bedrosian T, Galan A, Vaughn C, Weil ZM, Nelson RJ (2013). Light at night alters daily patterns of cortisol and clock proteins in female Siberian hamsters. J. Neuroendocrinol..

[27] Touzot M (2020). Artificial light at night alters the sexual behaviour and fertilisation success of the common toad. Environ. Pollut..

[28] de Jong M (2015). Effects of nocturnal illumination on life-history decisions and fitness in two wild songbird species. Philos. Trans. R. Soc. B.

[29] Spoelstra K (2015). Experimental illumination of natural habitat: An experimental set-up to assess the direct and indirect ecological consequences of artificial light of different spectral composition. Philos. Trans. R. Soc. Lond. B.

[30] Hattar S, Liao HW, Takao M, Berson DM, Yau KW (2002). Melanopsin-containing retinal ganglion cells: Architecture, projections, and intrinsic photosensitivity. Science.

[31] Gutman R, Dayan T, Levy O, Schubert I, Kronfeld-Schor N (2011). The effect of the lunar cycle on fecal cortisol metabolite levels and foraging ecology of nocturnally and diurnally active spiny mice. PLoS ONE.

[32] Dhairykar M, Singh KP, Kumar Jadav K, Rajput N (2020). Comparison of cortisol level in Asian elephants of different tiger reserves of Madhya Pradesh. Int. J. Vet. Sci. Anim. Husb..

[33] Sosnowski MJ, Benítez ME, Brosnan SF (2022). Endogenous cortisol correlates with performance under pressure on a working memory task in capuchin monkeys. Sci. Rep..

[34] Bewick V, Cheek L, Ball J (2004). Statistics review 12: survival analysis. Crit. care.

[35] Shkolnik, A. *Studies in the Comparative Biology of Israel's Two Species of Spiny Mice (genus Acomys)*. Hebrew (1966).

[36] Shkolnik A (1971). Diurnal activity in a small desert rodent. Int. J. Biometeorol..

[37] Levy O, Dayan T, Kronfeld-Schor N (2007). The relationship between the golden spiny mouse circadian system and its diurnal activity: An experimental field enclosures and laboratory study. Chronobiol. Int..

[38] Levy O, Dayan T, Kronfeld-Schor N (2011). Interspecific competition and torpor in golden spiny mice: Two sides of the energy-acquisition coin. Integr. Comp. Biol..

[39] Jones M, Dayan T (2000). Foraging behavior and microhabitat use by spiny mice, *Acomys cahirinus* and *A. russatus*, in the presence of Blanford's fox (Vulpes cana) odor. J. Chem. Ecol..

[40] Jones M, Mandelik Y, Dayan T (2001). Coexistence of temporally partitioned spiny mice: Roles of habitat structure and foraging behavior. Ecology.

[41] Kronfeld N, Dayan T, Zisapel N, Haim A (1994). Coexisting populations of *Acomys cahirinus* and *A. russatus*: A preliminary report. Isr. J. Zool..

[42] Kronfeld-Schor N, Dayan T (2003). Partitioning of time as an ecological resource. Annu. Rev. Ecol. Evol. Syst..

[43] Kronfeld-Schor N, Dayan T (1999). The dietary basis for temporal partitioning: Food habits of coexisting *Acomys* species. Oecologia.

[44] Pinter-Wollman N, Dayan T, Eilam D, Kronfeld-Schor N (2006). Can aggression be the force driving temporal separation between competing common and golden spiny mice?. J. Mammal..

[45] Shargal E, Kronfeld-Schor N, Dayan T (2000). Population biology and spatial relationships of coexisting spiny mice (Acomys) in Israel. J. Mammal..

[46] Pasco R, Gardner DK, Walker DW, Dickinson H (2012). A superovulation protocol for the spiny mouse (Acomys cahirinus). Reprod. Fertil. Dev..

[47] Lee TE, Watkins JF, Cash CG (1998). Acomys russatus. Mammal. Species.

[48] Dominoni D, Quetting M, Partecke J (2013). Artificial light at night advances avian reproductive physiology. Proc. R. Soc. B.

[49] Kempenaers B, Borgström P, Loës P, Schlicht E, Valcu M (2010). Artificial night lighting affects dawn song, extra-pair siring success, and lay date in songbirds. Curr. Biol..

[50] Le Tallec T, Théry M, Perret M (2016). Melatonin concentrations and timing of seasonal reproduction in male mouse lemurs (Microcebus murinus) exposed to light pollution. J. Mammal..

[51] Vonshak M, Dayan T, Kronfeld-Schor N (2009). Arthropods as a prey resource: Patterns of diel, seasonal, and spatial availability. J. Arid Environ..

[52] Levy O, Dayan T, Kronfeld-Schor N (2011). Adaptive thermoregulation in golden spiny mice: The influence of season and food availability on body temperature. Physiol. Biochem. Zool..

[53] Levy O, Dayan T, Rotics S, Kronfeld-Schor N (2012). Foraging sequence, energy intake and torpor: An individual-based field study of energy balancing in desert golden spiny mice. Ecol. Lett..

[54] Katz N, Dayan T, Kronfeld-Schor N (2018). Fitness effects of interspecific competition between two species of desert rodents. Zoology.

[55] Brzezinski A (1997). Melatonin in humans. N. Engl. J. Med..

[56] Hastings M, Vance G, Maywood E (1989). Some reflections on the phylogeny and function of the pineal. Experientia.

[57] Oster H (2006). The circadian rhythm of glucocorticoids is regulated by a gating mechanism residing in the adrenal cortical clock. Cell Metab..

[58] Mora F, Segovia G, Del Arco A, de Blas M, Garrido P (2012). Stress, neurotransmitters, corticosterone and body–brain integration. Brain Res..

[59] Farrell MR (2013). Sex Differences and Stress Effects in Corticolimbic Structure and Function.

[60] Son GH, Chung S, Kim K (2011). The adrenal peripheral clock: Glucocorticoid and the circadian timing system. Front. Neuroendocrinol..

[61] Schradin C (2008). Seasonal changes in testosterone and corticosterone levels in four social classes of a desert dwelling sociable rodent. Horm. Behav..

[62] Zatra Y (2018). Seasonal changes in plasma testosterone and cortisol suggest an androgen mediated regulation of the pituitary adrenal axis in the Tarabul’s gerbil Gerbillus tarabuli (Thomas, 1902). Gen. Comp. Endocrinol..

[63] Richardson CS, Heeren T, Kunz TH (2018). Seasonal and sexual variation in metabolism, thermoregulation, and hormones in the big brown bat (Eptesicus fuscus). Physiol. Biochem. Zool..

[64] Touitou S, Heistermann M, Schülke O, Ostner J (2021). Triiodothyronine and cortisol levels in the face of energetic challenges from reproduction, thermoregulation and food intake in female macaques. Horm. Behav..

[65] Rotics S, Dayan T, Kronfeld-Schor N (2011). Effect of artificial night lighting on temporally partitioned spiny mice. J. Mammal..

[66] Rotics S, Dayan T, Levy O, Kronfeld-Schor N (2011). Light masking in the field: An experiment with nocturnal and diurnal spiny mice under semi-natural field conditions. Chronobiol. Int..

[67] Padgett DA, Glaser R (2003). How stress influences the immune response. Trends Immunol..

[68] Khansari DN, Murgo AJ, Faith RE (1990). Effects of stress on the immune system. Immunol. Today.

[69] Zozaya SM, Alford RA, Schwarzkopf L (2015). Invasive house geckos are more willing to use artificial lights than are native geckos. Austral. Ecol..

[70] Komine H, Koike S, Schwarzkopf L (2020). Impacts of artificial light on food intake in invasive toads. Sci. Rep..

[71] Murphy S, Vyas D, Sher A, Grenis K (2022). Light pollution affects invasive and native plant traits important to plant competition and herbivorous insects. Biol. Invasions.

[72] Murphy SM (2021). Streetlights positively affect the presence of an invasive grass species. Ecol. Evol..

