# Supplementary material for: Fitness consequences of chronic exposure to different light pollution wavelengths in nocturnal and diurnal rodents
Source: Sci Rep. 2022 Oct 1;12:16486. doi: 10.1038/s41598-022-19805-1 (PMC9526750; doi:10.1038/s41598-022-19805-1)
Supplement: Supplementary file 1 — Supplementary Information. [file 41598_2022_19805_MOESM1_ESM.docx]

**Suplimentry materials:**

Table S1. Sample size of cortisol across season for A. russatus.

|  |  | Light treatment | | | |  |
| --- | --- | --- | --- | --- | --- | --- |
|  |  | Control | Blue | Yellow | White |  |
| 1. *russatus* | Mar-19 | 10 | 9 | 6 | 9 |  |
|  | Jun-19 | 7 | 4 | 4 | 7 |  |
|  | Dec-20 | 11 | 8 | 11 | 10 |  |

Table S2. Total number of newborns and females on consecutive years as a factor of season and light treatment.

|  | Pup/Female | Season | Control | Blue | White | Yellow | *df* | test | Prop>Chi square | Total number of pups |
| --- | --- | --- | --- | --- | --- | --- | --- | --- | --- | --- |
| *A. cahirinus* | Pups | Winter | 1 | 10 | 5 | 8 | 3 | Likelihood Ratio | <.0001 | 58 |
|  |  | Summer | 3 | 19 | 9 | 3 |  |  |  |  |
|  | Females | Winter | 13 | 16 | 17 | 15 | 3 |  |  |  |
|  |  | Summer | 11 | 10 | 5 | 7 |  |  |  |  |
| *A. russatus* | Pups | Winter | 0 | 1 | 4 | 0 | 3 | Likelihood Ratio | 0.0004 | 92 |
|  |  | Summer | 41 | 12 | 13 | 21 |  |  |  |  |
|  | Females | Winter | 14 | 13 | 17 | 15 | 3 |  |  |  |
|  |  | Summer | 10 | 12 | 5 | 7 |  |  |  |  |


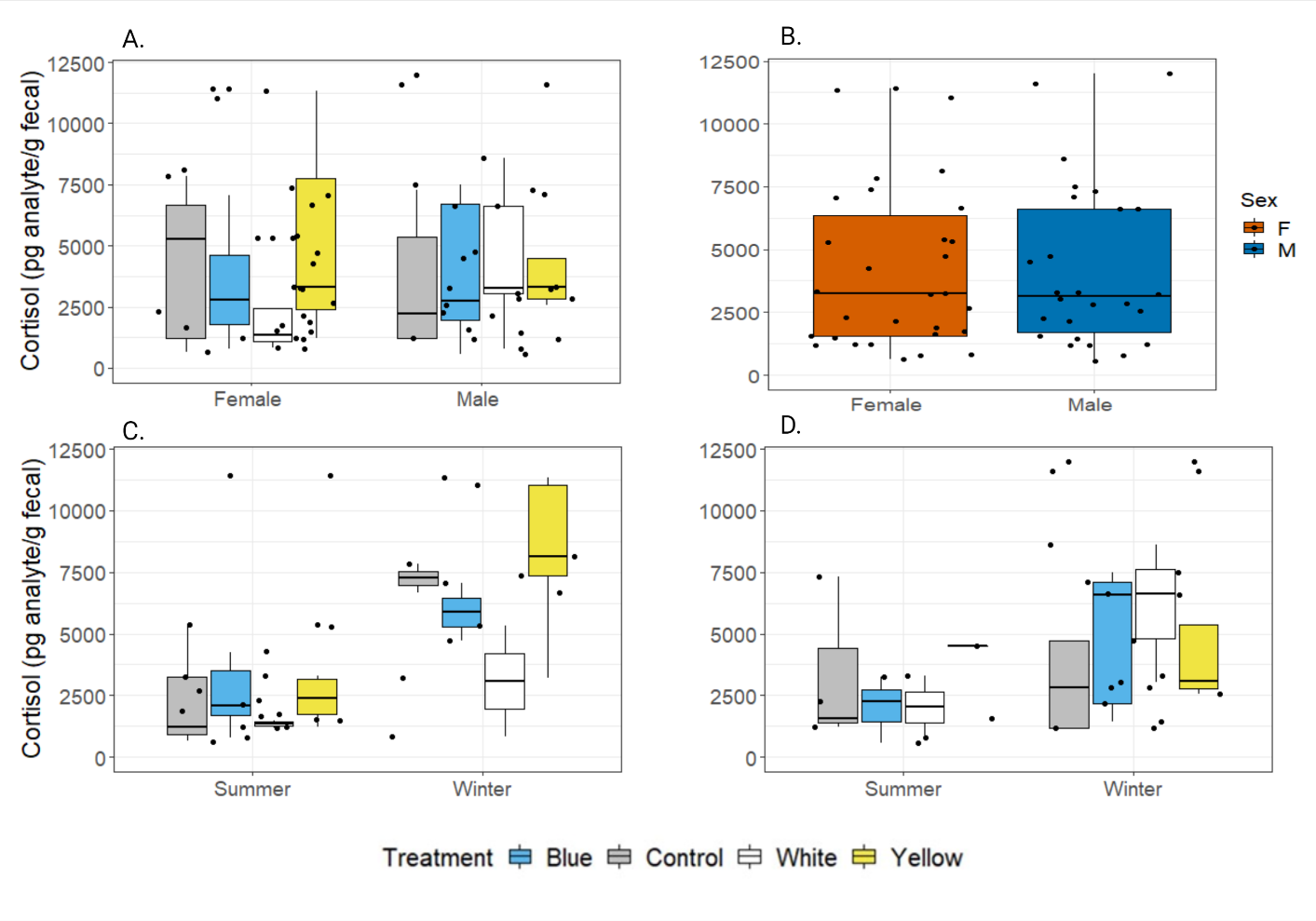


Figure S1. Fecal cortisol concentration of *A. cahirinus* exposed to the different treatments. (A) Cortisol concentration in females and males at winter and summer; (B) Cortisol difference between sexes in all *A. cahirinus* combined; (C) Cortisol concentration in females in summer and winter; (D) cortisol concentration in males in summer and winter. No significant changes in fecal cortisol levels between males and females, treatments, or seasons.
